# Adaptive evolution and functional constraint at TLR4 during the secondary aquatic adaptation and diversification of cetaceans

**DOI:** 10.1186/1471-2148-12-39

**Published:** 2012-03-24

**Authors:** Tong Shen, Shixia Xu, Xiaohong Wang, Wenhua Yu, Kaiya Zhou, Guang Yang

**Affiliations:** 1 Jiangsu Key Laboratory for Biodiversity and Biotechnology, College of Life Sciences, Nanjing Normal University, Nanjing, China

## Abstract

**Background:**

Cetaceans (whales, dolphins and porpoises) are a group of adapted marine mammals with an enigmatic history of transition from terrestrial to full aquatic habitat and rapid radiation in waters around the world. Throughout this evolution, the pathogen stress-response proteins must have faced challenges from the dramatic change of environmental pathogens in the completely different ecological niches cetaceans occupied. For this reason, cetaceans could be one of the most ideal candidate taxa for studying evolutionary process and associated driving mechanism of vertebrate innate immune systems such as Toll-like receptors (TLRs), which are located at the direct interface between the host and the microbial environment, act at the first line in recognizing specific conserved components of microorganisms, and translate them rapidly into a defense reaction.

**Results:**

We used TLR4 as an example to test whether this traditionally regarded pattern recognition receptor molecule was driven by positive selection across cetacean evolutionary history. Overall, the lineage-specific selection test showed that the *dN/dS *(ω) values along most (30 out of 33) examined cetartiodactylan lineages were less than 1, suggesting a common effect of functional constraint. However, some specific codons made radical changes, fell adjacent to the residues interacting with lipopolysaccharides (LPS), and showed parallel evolution between independent lineages, suggesting that TLR4 was under positive selection. Especially, strong signatures of adaptive evolution on TLR4 were identified in two periods, one corresponding to the early evolutionary transition of the terrestrial ancestors of cetaceans from land to semi-aquatic (represented by the branch leading to whale + hippo) and from semi-aquatic to full aquatic (represented by the ancestral branch leading to cetaceans) habitat, and the other to the rapid diversification and radiation of oceanic dolphins.

**Conclusions:**

This is the first study thus far to characterize the TLR gene in cetaceans. Our data present evidences that cetacean TLR4 has undergone adaptive evolution against the background of purifying selection in response to the secondary aquatic adaptation and rapid diversification in the sea. It is suggested that microbial pathogens in different environments are important factors that promote adaptive changes at cetacean TLR4 and new functions of some amino acid sites specialized for recognizing pathogens in dramatically contrasted environments to enhance the fitness for the adaptation and survival of cetaceans.

## Background

Microbial pathogens (bacteria, fungi, protozoa, and viruses) affect plants and animals of the world dramatically, including their survival, growth, development, and reproduction. In response to pathogen invasion, multicellular organisms have evolved several distinct immune-recognition systems. Unlike the adaptive immune system only found in vertebrates, the innate immune system is a universal and evolutionarily ancient mechanism existing in all multicellular organisms [1]. The innate immune system nonspecifically recognizes and kills pathogens at the first time and at the first line. The targets of innate immune recognition are called pathogen-associated molecular patterns (PAMPs), produced only by microbes and shared by a class of microorganisms. PAMPs are highly conserved because such molecular patterns are essential to the integrity, function, or replication of microbes [2]. Accordingly, PAMPs are recognized by a variety of host receptors called pattern recognition receptors (PRRs).

Toll-like receptors (TLRs) are among the best characterized PRRs that lie directly at the host-pathogen interface. Although TLRs have been regarded for a long time as a classic example of strong evolutionary conservation and intense functional constraint [3,4], a recent comparison of several *Drosophila *genomes showed for the first time the fast evolution between closely related species [5]. Although this contradicts the traditional view regarding innate immunity, this finding is congruent with theoretical prediction that over evolutionary time TLRs may be engaged in co-evolutionary arms races with their microbial ligands. Some recent discoveries and characterization surveys of TLRs variation in vertebrates [5-7] provide further corroboration for this prediction. To date, however, very few studies have been conducted on the evolution of TLRs in a limited number of vertebrate species, including primates [3,8-10], ungulates [11], birds [12,13], and bony fishes [14]. Furthermore, the results from different studies are incongruent with or contradict each other. For example, although Ferrer-Admetlla et al. [6] regarded balancing selection as the best explanation for sequence variation at human TLRs, Mukherjee et al. [3] did not detect any effect of natural selection on TLRs of the Indian population and thus supported the traditional viewpoint that purifying selection is the major driving force for the evolution of TLRs. In some inter-specific studies, Ortiz et al. [15] detected positive selection at the TLRs of five primate species only, whereas Nakajima et al. [8] found the action of positive selection on TLR4 when they examined a more extensive phylogenetic sampling. Recently, Wlasiuk et al. [9] and Wlasiuk and Nachman [10] detected positive selection on most TLR loci of primates, but intra-specific polymorphisms were found to be influenced mainly by population demography rather than by adaptive evolution. In other words, they found that primate TLRs are characterized by a mode of episodic evolution. Positive selection and evolutionary constraint have also been detected in birds [13] and bony fishes [14], suggesting the role of adaptive evolution in response to changes of environmental pathogens. Considering the limited number of taxa and loci examined in these studies, a clear picture of the evolution of the TLR gene family has not been painted so far, and more data are necessary to resolve this problem.

Cetaceans, including whales, dolphins, and porpoises, are a group of secondarily adapted marine mammals with a history of transition from terrestrial (land) to full aquatic habitats and subsequent adaptive radiation in waters around the world. Although the exact origin and evolutionary history of extant cetaceans remains unclear, a widely accepted view is that the direct terrestrial ancestors of cetaceans (a group of mammals called artiodactyls [16,17]) returned to the sea around 50 MYA[18-21]. The ancient cetaceans evolved gradually to conquer nearly all oceans and some rivers of the world [22-24], and finally diversified into a group of fully aquatic mammals including nearly 85 extant species that can be subdivided into two suborders (Odontoceti and Mysticeti) [25-27]. During the transition from land to sea and the radiation and diversification into various aquatic environments, cetaceans must have been confronted with formidable challenges from ever-changing environmental pathogens. For this reason, cetaceans could be one of the most ideal candidate taxa for studying the evolutionary process and the associated driving mechanisms of vertebrate innate immune systems such as TLRs.

Here, TLR4 was used as an example to reveal the evolutionary history of pattern recognition molecules across cetaceans and their closest terrestrial relatives. TLR4 is expressed on the cell membrane and is mainly responsible for the recognition of lipopolysaccharides (LPS) from Gram-negative bacteria [28] and even components of yeast, *Trypanosoma*, and viruses [29]. This molecule interacts with LPS indirectly aided with myeloid differentiation factor 2 (MD-2) [30] through the formation of a duplex heterodimer (TLR4-MD-2-LPS)_2 _that is essential to activate a signaling pathway mediating the defense against Gram-negative bacteria. It has been reported that some substitutions in the changed amino acid residues of TLR4 can alter the interaction among TLR4, MD-2, and LPS, and modify the TLR4/MD-2 immunological responses [10,13]. In this study, the open reading frames (ORF) of TLR4 from representative cetaceans and some closely related artiodactylans were sequenced to elucidate whether this innate immune gene has been the target of positive selection in cetacean evolutionary history. The aims of this study were 1) to find evidence of positive selection at TLR4 in cetacean origin and evolution, and 2) to evaluate whether the evolutionary rate of TLR4 varied in different cetacean lineages, and if so, what factors could account for this evolutionary pattern. It was interesting to find compelling evidence of positive selection acting on TLR4 throughout cetacean evolution, from their origin till the present, and it was speculated that the species-specific effects and/or the complex interaction of multiple factors (abiotic and biotic) might have played a major role in driving the heterogeneity in the evolutionary rate of cetacean TLR4.

## Results

**Figure 1:**
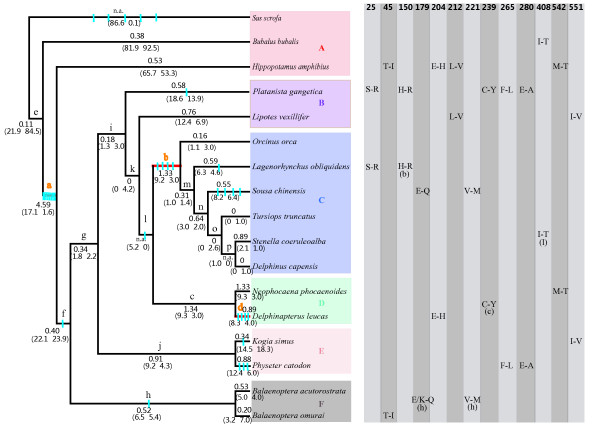
**Positive selection at TLR4 across the cetacean phylogeny**. Branches **a **to **p **correspond to those in supplementary Table S2. The ω value calculated by the free-ratio model is labeled along each branch. In some cases, zero synonymous substitutions lead to a ω value of infinity (n.a.). The estimated numbers of nonsynonymous and synonymous changes are shown in parentheses. The branches in red show strong evidence of undergoing positive selection. Amino acid changes were estimated by parsimony method, and every substitution of these sites is marked in blue. Six clades in which amino acid substitution occurred are filled with six different colors. The parallel amino acid changes are listed on the right of the corresponding terminal branches, while **b, c, h**, and **l **in parentheses stand for the internal branches on which parallel changes occurred. Amino acid positions (numbers) and parallel changes at each position were listed in the right part of the figure1. A = even-toed ungulates, B = river dolphins, C = oceanic dolphins, D = porpoises and white whales, E = sperm whales, F = baleen whales.

In this study, the full sequences containing 2250 bp of TLR4 open reading frame (ORF) from 17 representative cetaceans and three even-toed ungulates were obtained, 12 of which were newly determined and have been deposited in GenBank with accession nos. JN642608-JN642619 (Additional file 1: Table S1). The Bayesian analyses and Neighbor-Joining (NJ) method yielded a similar topology (Figure 1), which is basically consistent with a widely accepted hypothesis of whale phylogeny [17,31-33]. This phylogeny was then used as the working topology in the subsequent analyses. To our knowledge, this is the first study thus far to characterize a TLR locus in cetaceans and to provide some novel insights into the evolution of the innate immune system in the cetacean clade.

### Positive selection at cetacean TLR4

The site model incorporated in Phylogenetic Analysis by Maximum Likelihood (PAML) was used to reveal whether cetacean TLR4 was subjected to positive selection. We compared nested models and found that a model including sites with ω > 1 fitted the data significantly better than did a neutral model. Model M8 detected 25 (3.3%) sites under selection with the average ω value of 3.55 in cetacean (Table 1). The specific codons identified by the Bayes empirical Bayes (BEB) approach with a posterior probability of 90% constituted an even smaller fraction (11 codons, 1.5%). With the use of Datamonkey, 17 and 13 codons were detected by fixed effects likelihood (FEL) and random effects likelihood (REL), respectively, whereas no site was detected by single likelihood ancestor counting (SLAC). When all these analyses from PAML and Datamonkey were combined, nine codons (150, 179, 183, 207, 228, 247, 272, 280, and 324) were picked out as robust sites under positive selection by at least two Maximum Likelihood (ML) methods, five (179, 207, 228, 272, 280) of which were predicted by three ML methods. In general, the more radical the amino acid substitutions are, the more likely they will affect function during evolution [34]. Most of the nine codons identified under selection made relatively conservative changes, while sites 272 and 280 were involved in radical changes in their physicochemical properties (size, polarity, and electric charge). In particular, codon 280 showed the strongest evidence of selection not only because it was detected by three ML methods, but also because it showed radical changes in three independent lineages (Table 2).

**Table 1:** Tests for positive selection at cetacean TLR4 using branch model and site models

Model	Models Compared	-2lnΔL	df	*p *value	Proportion of Sites under Selection	ω (*dN/dS*) of Sites under Selection
Site model	M1 versus M2	16.10	2	< 0.0001	0.033	3.45

	M7 versus M8	16.48	2	< 0.0001	0.033	3.55

	M8a versus M8	16.10	1	< 0.0001		

Branch model	M0 versus full	44.13	31	0.0595		

**Table 2:** Positive selection at amino acid sites of cetacean TLR4

**AA Positions**^**a**^	PAML Site Model (M8) *p *> 0.9	**PAML Branch-Site Model**^**c**^	FEL^d ^*p *< 0.2	REL^d ^BF > 50	AA Changes	Parallel Changes	**Property Changes**^**e**^	Protein Domain	**Functional Information**^**f**^	**Clade**^**g**^
25					Ser-Arg	Yes	SM, P, NEU-P.POS	LRR3		B, C
28			0.07		Leu-Trp		NP, NEU-P, NEU	LRR3		A, D
					Leu-Pro		NP, NEU-SM, NP, NEU			
45			0.16		Thr-Ile	Yes	SM, P, NEU-NP, NEU	LRR4		A, F
104			0.17		Leu-Val		NP, NEU-NP, NEU	LRR6		
					Leu-Ser		NP, NEU-SM, P, NEU			
128		0.875			Glu-Pro		P, NEG-SM, NP, NEU	LRR7		D
133		0.723			Asn-Lys		SM, P, NEU-P, POS	LRR7		G
139		0.708			Gly-Glu		SM, NP, NEU-P, NEG	LRR8	Adjacent to site involved in interaction with MD2	G
149		0.565			Ser/Leu -Thr		SM, P, NEU/NP, NEU -SM, P, NEU	LRR8		G
**150**	0.995			228.23	His-Arg	Yes	P, POS-P, POS	LRR8		A, B, C
					His-Asp		P, POS-SM, P, NEG			
177				61.94	Asn-Thr Asn/Thr -Ile Asn-Lys Ile-Asn		SM, P, NEU-SM, P, NEU SM, P, NEU/SM, P, NEU -NP, NEU SM, P, NEU-P, POS NP, NEU-SM, P, NEU	LRR9	Adjacent to site involved in ligand binding and interaction with MD2	A, C, G
**179**	0.992		0.07	647.96	Lys-Glu Glu-Gln Glu/Lys- Gln	Yes	P, POS-P, NEG P, NEG-P, NEU P, NEG/P, POS-P, NEU	LRR9		A, C, F
**183**			0.12	51.06	Arg-Ser Arg-Thr		P, POS-SM, P, NEU P, POS-SM, P, NEU	LRR9		C, D
204					Glu-His	Yes	P, NEG-P, POS	LRR10		A, D
**207**	1.000		0.08	1563.58	Gly/Lys -Arg Arg-Lys Arg-Thr Lys-Arg		SM, NP, NEU/P, POS -P, POS P, POS-P, POSP, POS-SM, P, NEU P, POS-P, POS	LRR10		A, G, C, E
212					Leu-Val	Yes	P, POS-NP, NEU	LRR10		A, B
221			0.1		Val-Met	Yes	NP, NEU-NP, NEU	LRR11		C, D, F
**228**	0.994		0.15	544.88	Asp/Ser/Cys -Asn Asp-Asn		SM, P, NEG/SM, P, NEU/SM, NP, NEU -SM, P, NEU SM, P, NEG-SM, P, NEU	LRR11		A, G
230	0.978				Gly/Glu/Asp -Arg Asp-His		SM, NP, NEU/P, NEG/SM, P, NEG -P.POS SM, P, NEG-P, POS	LRR11		A, E
239				50.32	Cys-Tyr	Yes	SM, NP, NEU-P, NEU	LRR12		B, D, G
**247**			0.14	86.14	Ile-Thr Thr-Ile		NP, NEU-SM, P, NEU SM, P, NEU-NP, NEU	LRR12	Adjacent to site involved in interaction with ligand binding	C, G
*250*^b^	0.936				Asp/Ala -Lys Asp/Lys/Ala -Asn Asn-Lys		SM, P, NEG/SM, NP, NEU -P, POS SM, P, NEG/P, POS/SM, NP, NEU -SM, P, NEU SM, P, NEU-P, POS	LRR12	Ligand binding	A, E, G
265					Phe-Leu	Yes	NP, NEU-NP, NEU	LRR13		B, E
**272**	0.997		0.13	188.28	Gly/Asp- His Gly-His His-Gly		SM, NP, NEU/SM, P, NEG-P, POS SM, NP, NEU-P, POS P, POS-SM, NP, NEU	LRR13	Adjacent to site involved in interaction with ligand binding	A, C
**280**	0.952		0.18	191.07	Glu-Ala Gln/Glu- Ala	Yes	P, NEG-SM, NP, NEU P, NEU/P, NEG-SM, NP, NEU	LRR13		A, B, E
302		0.624			His-Arg		P, POS-P.POS	LRR14		D
304				55.05	Asp-Asn Asn-Pro		SM, P, NEG-SM, P, NEU SM, P, NEU-SM, NP, NEU	LRR14		G
**324**	0.996			301.87	Asn-Ser Asn-Lys Gly-Asn		SM, P, NEU-SM, P, NEU SM, P, NEU-P, POS SM, NP, NEU-SM, P, NEU	LRR15	Adjacent to site involved in interaction with ligand binding (hydrogen bond)	C, E, G
342				53.56	Asn-Ser Asn/Ser- Thr		SM, P, NEU-SM, P, NEU SM, P, NEU/SM, P, NEU-SM, P, NEU	LRR16	Adjacent to site involved in interaction with ligand binding (hydrogen bond)	A
351				0.17	Ile/Ala-Val		NP, NEU/SM, NP, NEU-NP, NEU	LRR16	Adjacent to site involved in interaction with ligand binding (hydrophobic interaction)	G
368		0.576			Ile-Thr		NP, NEU-SM, P, NEU	LRR17	Adjacent to site involved in interaction with ligand binding (hydrophobic interaction)	G
404				0.08	Leu-Met		NP, NEU-NP, NEU	LRR18		C
408					Ile-Thr	Yes	NP, NEU-SM, P, NEU	LRR19		A, G
409				0.19	Leu/Ile/Phe -Val		NP, NEU/NP, NEU/NP, NEU -NP, NEU	LRR19		A
482				0.16	Ser/Trp- Phe Phe/Ser/Trp -Leu		SM, P, NEU/P, NEU-NP, NEU NP, NEU/SM, P, NEU/P, NEU -NP, NEU	LRR22		A
542	0.903				Met-Thr	Yes	NP, NEU-SM, P, NEU	LRRCT		A, D
551	0.938				Ile-Val Val-Ile	Yes	NP, NEU-NP, NEU NP, NEU-NP, NEU	Transmembrane		B, F
559				0.16	Val-Ala		NP, NEU-SM, NP, NEU	Transmembrane		G
690		0.564			Arg-Gln		P, POS-P, NEU	TIR		D
740		0.790			Glu-Asp		P, NEG-SM, P, NEG	TIR		G
742		0.697			Asn-Arg		SM, P, NEU-P, POS	TIR		G
743				0.18	Gln-Glu		P, NEU-P, NEG	TIR		A, F

^a ^Codons identified by more than one ML method were in bold and underlined.

^b ^Site 250 in italic was mapped onto the 3D structure of TLR4, since it directly participates in binding of LPS to TLR4.

^c ^Codons were identified by branch-site model in PAML. Details were in Materials and Methods and Additional file 2: Table S2.

^d ^Codons were estimated in DATAMONKEY.

^e ^SM, small; NP, nonpolar; P, polar; NEU, neutral; POS, positively charged; NEG, negatively charged.

^f ^Codons were in the functional regions predicted by the three-dimensional structure in Shishido et al. 2010. LRR = Leucine-rich repeat, CT = C-terminal, TIR = cytoplasmic Toll/IL-1 receptor

^g ^Amino acid substitutions occurred in the following clades: A = even-toed ungulates, B = river dolphins, C = oceanic dolpins, D = porpoises and white whales, E = sperm whales, F = baleen whales, G = more than one equally parsimonious reconstruction

The amino acid changes reconstructed by parsimony were distributed along 42% of examined cetartiodactylan branches or 46% of examined cetacean branches. Thirteen codons (25, 45, 150, 179, 204, 212, 221, 239, 265, 280, 408, 542, and 551) showed parallel amino acid changes (Table 2), which could be regarded as candidates under selection. These codons were scattered across the entire whale phylogeny (Figure 1), rather than accumulated in just some specific lineages.

The LRT tests based on the branch model suggested that the free-ratio model fitted the data better than did the one-ratio model (Table 1), indicating that *dN/d*S ratios were indeed different among lineages. The ω values along three branches were found to be greater than 1 with nearly significant statistical support (*p *= 0.0595): branch *a *leading to the last common ancestor of cetaceans and hippos (ω = 4.59), branch *b *leading to oceanic dolphins (ω = 1.33), and branch *c *leading to the last common ancestor of Phocoenidae (porpoises) + Monodontidae (white whales) (ω = 1.34) (Figure 1). For all the cetacean lineages examined, ω values ranged from 0.0001 to 1.34, with an average of 0.61 (Figure 1).

When we used the branch-site model to predict positive selection acting on each branch (Additional file 2: Table S2), two lineages were detected under positive selection because likelihood ratio test (LRT) tests suggested that model A fitted the data better than did model M1a along branches *a *(whale + hippo) (LRT of test 2 = 5.40, df = 1, *p *= 0.02) and *d *(beluga whale) (LRT of test 2 = 8.20, df = 1, *p *= 0.004) (Figure 1). Six and three codons were respectively detected under positive selection along these two branches (Additional file 2: Table S2). The BEB values of the positively selected sites along these two branches were not high (0.564 <*p *< 0.875), which is not surprising, however, as suggested by Zhang et al. [35]. Of these positively selected codons identified using the branch-site model, sites 139 (*p *= 0.708) in branch *a *(whale + hippo) and 128 (*p *= 0.875) in branch *d *(beluga whale) (Figure 1) showed a stronger signature, with radical amino acid changes in size, polarity, and electric charge (Table 2), and fell in the functionally important region of TLR4 as suggested by Shishido et al. [36].

### Positive selection at different functional domains and 3D structure of cetacean TLR4

**Figure 2:**
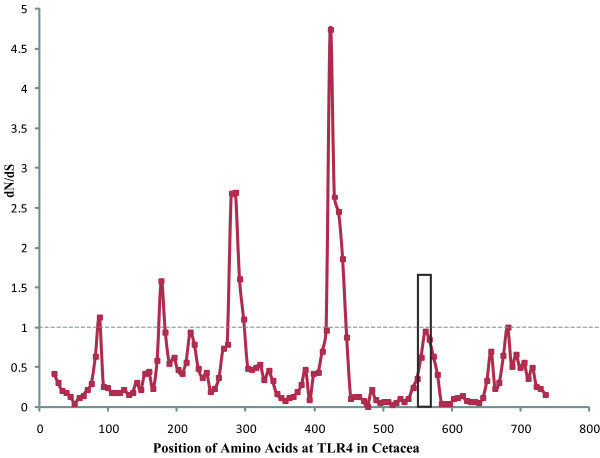
**Average ω ratio of a 20-codon sliding window along cetacean TLR4 protein sequences**. High values (ω > 1) indicate positive selection, whereas low values (ω < 1) indicate purifying selection. The black box indicates the transmembrane domain.

**Figure 3:**
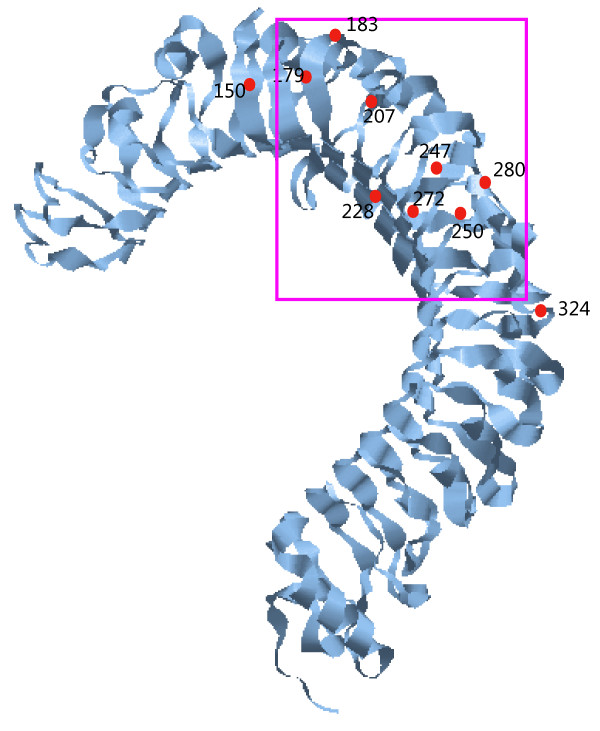
**Distribution of positively selected codons in the three-dimensional structure of cetacean TLR4**. The area important for ligand binding is squared in pink.

The average rate of cetacean TLR4 evolution was 0.61 as inferred with PAML M0. Where domain-specific ω values are concerned, the transmembrane domain (TM) domain had a higher ω value (ω = 2.17) than did the other two domains (ω = 0.66 for extracellular domain (EXT) and 0.31 for cytoplasmic domain (CY)). However, sliding window analysis (Figure 2) and the above ML methods showed that most codons under positive selection were located within the EXT domain, with higher ω values scattered almost all over the leucine-rich repeat (LRR) regions of the EXT domain, particularly between AA80 and AA520. All tests showed that nonsynonymous substitutions were rarely located in the CY and TM domains, and all the sites identified by at least two ML methods (Table 2) fell in the EXT domain. When the amino acids under positive selection were mapped onto the crystallographic structure of TLR4, most of the positively selected sites were found to fall in the regions of interaction with LPS (Figure 3) within EXT. In addition, site 250 identified only by M8 was also mapped onto the region binding with LPS, which can be regarded as a weak support for the stronger selection on EXT (Figure 3).

### Association of ω values with group sizes

We tested whether the selection on TLR4 was correlated with group sizes of cetaceans derived from May-Collado et al. [37]. The ordinary linear regression analyses did not reveal a significant association between ω values and group sizes for all cetaceans (R^2 ^= 0.018, *p *= 0.641, df = 13). When delphinids were specially considered, a moderate to high R^2 ^value (R^2 ^= 0.710) was obtained but not supported with a statistical significance (*p *= 0.158, df = 5).

## Discussion

### Strong adaptive evolution of TLR4 during the habitat shift from land to water

The present study revealed that the branch leading to whale + hippo was under the strongest positive selection at TLR4, evidenced by the highest ω value (4.59, *p *= 0.02) and the maximum number of specific codons (n = 9) detected by branch site model (Figure 1 and Additional file 2: Table S2). This lineage was just before the differentiation between cetacean and hippo, both of which are regarded to share a common semi-aquatic ancestor that branched off from other artiodactyls [38]. In other words, this lineage represents the habitat transition of the terrestrial ancestors of cetaceans from land to semi-aquatic habitat. It is clear that pathogens were dramatically different in terms of diversity and abundance between land and water. Therefore, in such a phase of habitat shift, TLR4, which interacted directly with environmental pathogenic microbes, must have been subjected to strong selective pressures. Moreover, a signal of positive selection was also detected in the lineage leading to the common ancestor of cetaceans (branch *f *in Figure 1). This lineage represents the early evolutionary history of cetaceans from semi-aquatic to full aquatic (marine) habitat, during which the cetaceans were faced with the challenges of infectious pathogens in changing habitats. Although the ω value of this branch was less than 1 (0.4), one positively selected codon (AA324) was identified, which caused radical amino acid change from a nonpolar Gly to a polar Asn. That is to say, TLR4 must have adaptively modified to recognize and bind potential novel pathogens in the new environment, which is again in accordance with the expectation of the co-evolution arms race model.

### Adaptive evolution of TLR4 associated with rapid diversification of oceanic dolphins

Another strong signature of positive selection was detected along the lineage leading to oceanic dolphins, i.e., the family Delphinidae (delphinids). Four (150H-R, 179 K-E, 272 G-H, 324 N-S) adaptive AA changes were found on this lineage with a ω value of 1.33. In particular, site 272 in oceanic dolphins was identified by three ML methods and constituted the most radical change from small, nonpolar, and neutral Gly to polar and positively charged His (Table 2).

The stronger level of positive selection on this lineage might have resulted from the rapid diversification and adaptive radiation that this group has experienced. Molecular phylogenetic studies [24,32,33,39] have suggested that a rapid radiation and diversification that occurred near the Miocene/Pliocene boundary. The delphinid clade has been the most speciose living group of Cetacea [25] (containing 35 of 89 known species) and the most ecologically versatile, occupying tropical to polar latitudes, coastal and oceanic waters, estuaries, and sometimes freshwater rivers. In response to the dramatic changes in the prevalence, intensity, virulence, and diversity of microbial pathogens in various aquatic environments, innate immune genes such as TLR4, as expected, had to make evolutionarily adaptive changes that were necessary to ensure the long-term survival and successful radiation of dolphins and porpoises in the sea.

### Domain-specific selective pressure

Of the three functional domains of TLR molecules, the EXT domain is at the first line of defense against invasive pathogens and plays a key role in directly recognizing and binding PAMPs such as LPS from Gram-negative bacteria [40]. According to the hypothesis of an arms race between pathogens and vertebrate immune systems, it is reasonable to find a stronger effect of positive selection in the EXT domain than in the TM and CY domains. This was corroborated by most codons under positive selection being located within this region and the predominant higher codon-specific ω values being scattered in the LRR region of the EXT domain. In particular, most sites under positive selection were found to fall in EXT regions interacting with LPS (Figure 3), which is similar to that found in primate TLR4 [10].

It is somewhat surprising, however, that the overall ω value in the TM region (2.1712) is much higher than those in the CY (0.3131) and the EXT (0.6613) domains. Actually, this is not a novel finding of this study. A similar phenomenon was reported in primates [10] and ruminant [11], but no explanation was given. Nevertheless, it seems irrational to explain this strange higher ω value with a strong signature of positive selection, because only two sites in this region were identified as candidates under positive selection, although with only one ML method (Table 2). Sliding window analysis also verified that most codons with higher ω values > 1 were scattered in the EXT domain, whereas only very few of such codons were found in the TM and CY domains. Given that the TM domain was only 23 amino acids in length and only a very small number of candidate selective sites were identified with weak support, it is difficult to obtain an estimate with high statistical significance. The highest ω value in the TM domain, therefore, was most likely a biased estimate or an artifact.

### Species-specific pattern of positive selection

Evolutionary analysis of cetacean TLR4 revealed an inconstant pattern of positive selection across the cetacean phylogeny, with different species of extant cetaceans (terminal branches in Figure 1) displaying contrasted selective pressures (Figure 1). What factors triggered or correlated with heterogeneity in the evolutionary rate of cetacean TLR4 will be an interesting question to answer. To our knowledge, many life-history traits and species or population-level factors such as mating system, distribution area, habitat type, migration or dispersal pattern, and social structure, are different among cetacean species, and thus might have caused the variation in pathogen pressures and disease risks. To avoid the problem of uncertainty in these factors along the long branches, we focused only on the extant cetacean species (terminal branches in Figure 1). Unfortunately, at present, due to insufficient understanding of these factors for different cetacean species, it is not possible for us to address their relationships with heterogeneity in the evolutionary rate of cetacean TLR4 using quantitative association analyses. However, some preliminary direct comparisons between life-history traits or population-level factors and selective pressures suggest that a complex species-specific effect might have been an important mechanism to control the heterogeneity in the evolutionary rate of cetacean TLR4. For example, the two river dolphins examined in this study, namely, the Ganges river dolphin *Platanista gangetica *and the Yangtze river dolphin *Lipotes vexillifer*, both showed similarly lower ω values; however, two positively selective sites were identified in the former while no such site was detected in the latter. In addition, a representative species from the most inshore shallow waters (the Indo-Pacific humpback dolphin) showed four sites under positive selection, which might imply the negative anthropogenic impacts (direct or indirect) in coastal waters on the immune system. However, another species from coastal waters (the finless porpoise *Neophocaena phocaenoides*) did not display a similar enhanced selection over other offshore or oceanic species. Furthermore, some closely related species showed significantly contrasted levels of selection. For instance, oceanic dolphins within the family Delphinidae showed great divergence in evolutionary rates of TLR4, from nearly 0 (bottlenose dolphin and long-beaked common dolphin *Delphinus capensis*) to 0.89 (the striped dolphin *Stenella coeruleoalba*). Although there is a tendency of group size increasing in delphinoids [37], there seems to be no strong effect on the evolution of TLR4, because no significant association between group sizes and ω values was found not only for all cetaceans but only for delphinids. For this reason, it is necessary to further investigate this issue in the future, with an increasing uncovering of life history and population characteristics of different cetacean species, and a more comprehensive understanding of the molecular evolution of cetacean TLRs as well.

## Conclusions

In summary, our data presented in this study strongly suggest that TLR4 has undergone adaptive evolution against the background of purifying selection across cetacean enigmatic history of transition from land to full aquatic habitats and subsequent adaptive radiation in waters around the world. Most sites under positive selection were found to fall in the LRR region of the EXT domain interacting with LPS, which was accordance with the hypothesis of an arms race between pathogens and vertebrate immune systems. In addition, some preliminary direct comparisons between life-history traits or population-level factors and selective pressures suggest that a complex species-specific effect might have been an important mechanism to trigger the heterogeneity in the evolutionary rate of cetacean TLR4.

## Methods

### Samples and DNA sequencing

Total genomic DNA was extracted from muscle and blood samples from 11 cetacean species (Additional file 1: Table S1) and a hippopotamus (*Hippopotamus amphibius*) using Dneasy Blood & Tissue Kit (Qiagen) according to the manufacturer's instructions. This research is compliant with the "Animal Research: Reporting In Vivo Experiments" (ARRIVE) guidelines. Because these samples were collected from stranded or incidentally captured/killed animals in coastal China seas, ethical approval was not needed in such a situation. Voucher specimens were preserved at Nanjing Normal University. In addition, coding sequences of the sperm whale (*Physeter catodon*), killer whale (*Orcinus orca*), Pacific white-sided dolphin (*Lagenorhynchus obliquidens*), and water buffalo (*Bubalus bubalis*) were downloaded from GenBank with accession numbers AB500181, AB492857, AB492856 and HM469969, respectively, whereas the coding sequence of the pig (*Sus scrofa*) was retrieved from Ensemble Database with accession no. ENSSSCG00000005503.

To amplify the ORF region of TLR4, we designed a series of overlapping primers (Additional file 3: Table S3) in conserved ORF regions searched with ORF Finder http://www.ncbi.nlm.nih.gov/gorf/ in the bottlenose dolphin (*Tursiops truncatus*) (Ensemble GeneScaffold_1465), dog (*Canis familiaris*) (Ensemble Gene ID ENSCAFG00000003518), and water buffalo (GenBank accession no HM469969). PCR mixtures (30 μl) contained 0.2 μmol of each primer, 3 μl of 10× PCR buffer, 0.2 mmol of dNTP, 1 unit of Taq polymerase (Takara), and 0.8 μl of genomic DNA. The PCR condition was as follows: 95°C denaturation for 5 min, then running 35 cycles of 95°C 30 s, 55-58°C 30 s, 72°C 40 s, and 72°C elongation for 10 min. PCR products were purified using a Gel Extraction Kit (Promega) and sequenced in both directions using ABI PRISM 3730 DNA Sequencer.

### Statistical analysis

The specificity of these newly generated sequences was examined by comparison with the published nucleotide database at GenBank by BLAST (NCBI). Protein sequences were aligned using FASTA [41] and Muscle vs3.7 [42]. The nucleotide sequences and putative amino acid sequences were further aligned using MEGA4 [43]. Phylogenetic relationships were reconstructed using Bayesian inference (BI) in MrBayes 3.1.2 [44] and the NJ method in MEGA4. In Bayesian analysis, the WAG model [45] was selected using Modeltest [46]. Four Markov chains were run for 10^6 ^generations and were sampled every 100 generations to yield a posterior probability distribution of 10^4 ^trees. The first 2000 trees were discarded as burn-in. A three-dimensional (3D) domain structure of the cetacean TLR4 was predicted using CPHmodels-3.0 Server http://www.cbs.dtu.dk/services/CPHmodels/.

### Detections of positive selection

Comparisons of nonsynonymous/synonymous substitution ratios (ω = *dN/dS*) has become a useful means for quantifying the impact of natural selection on molecular evolution [47,48]. If ω = 1, amino acid substitutions may be largely neutral; ω > 1 is evidence of positive selection, whereas ω < 1 is consistent with purifying selection although the possibility of positive selection cannot be excluded in such a case.

However, the straightforward use of the ω ratio to detect positive selection, through direct calculation of *dN *and *dS *between sequences, has become rarely effective, because adaptive evolution most likely occurs at a few time points and at most times has an effect on only a few amino acids. In such cases, the ω ratio averaged over time and over sites will not be significantly > 1, even if adaptive molecular evolution may have occurred [49]. Thus, the codon-based maximum likelihood (CodeML) method in the PAML package [50] was used to detect lineage- or site-specific selection. Nested models were compared with critical values of the Chi square distribution using the LRT statistic (-2[LogLikelihood1 - LogLikelihood2]), and degrees of freedom as the difference in the number of parameters were estimated with each model. A model of codon frequencies, i.e. F3 × 4, was used for the present analyses. To check for convergence, all analyses were run twice, respectively using initial ω values of 0.5 and 1.5.

To evaluate positive selection on TLR4 across the presently examined cetacean species, we first used site models implemented in the CodeML program in PAML version 4.0 [50], not allowing variation among lineages. Models M1, M7, and M8a restricted sites with ω ≤ 1, whereas models M2 and M8 included a class of sites with ω > 1. The sites with a posterior probability > 0.9 were considered as candidates for selection. Then we used improved statistical methods in Datamonkey web server [51], which computed nonsynonymous and synonymous substitutions at each codon position to further evaluate the selection. Three ML methods with default settings applied in this web were used: SLAC, REL, and FEL. SLAC, which calculates the expected and observed numbers of synonymous and nonsynonymous substitutions to infer selection, is a conservative test. FEL directly estimates *dN *and *dS *based on a codon-substitution model, whereas REL, allowing the synonymous and nonsynonymous substitution rates to vary among codon sites [52], uses the Bayes factors to determine a site as selected. The default settings with significance levels of 0.1 for SLAC and 0.2 for FEL were used. Bayes factor > 50 for REL was implemented. Normally, REL is more powerful than SLAC and FEL, but it has the highest rate of false positives [52]. These three predictions were conducted using the HKY85 model, which is thought to perform well for a low number of sequences [13].

To detect the independent ω ratio for each branch of the tree, a free-ratio model was run with CodeML in PAML version 4, which allows each branch to have a separate *dN/dS *[50]. This involves as many ω parameters as the number of branches in the tree and is parameter-rich for a tree of many species, which is applicable only to a small data set [53].

Positive selection was further detected with the improved branch-site likelihood method as described in Zhang et al. [35]. This test appeared to be conservative overall, but exhibited better power than did the branch-based test. This is a simple modification to the branch-site model proposed by Yang and Nielsen [54] and was used to construct two new LRTs, referred to as test 1 and test 2. Test 1 is unable to reliably distinguish between positive selection and relaxed constraint on the foreground branches, whereas test 2 can accurately distinguish between them and thus often has stronger power than test 1 in detecting positive selection. It is worth noting that when positive selection operates episodically on a few amino acid sites, the signal may be masked by negative selection. Especially if positive selection has affected only one lineage or a very few lineages on the tree, the tested-positive selection at any single site may not be strong enough for the BEB probability to reach high levels. In this case, however, in this case, Zhang et al. [35] still suggested the use of this method to detect positive selection even if the affected sites cannot be reliably inferred.

The amino acid changes that occurred in the positively selected sites were inferred using maximum parsimony by Mesquite [55]. We marked the positively selective sites detected by more than one ML method (Table 2) and those detected by the branch-site model (Additional file 2: Table S2) onto the phylogenetic tree (Figure 1) to observe the distribution of these sites across cetacean phylogeny.

To further visualize variation of ω at TLR4 across cetacean phylogeny, we undertook a sliding window analysis using the software SWAAP1.0.2 [56], with window size at 60 bp (20 codons) and step size at 15 bp (5 codons). In addition, the ω value in each of three domains, i.e., the EXT, TM, and CY, was estimated using model M0 to evaluate the relative extent of functional constraint among these domains. The domains were identified with Motifscan http://myhits.isb-sib.ch/cgi-bin/motif_scan[57] and Simple Modular Architecture Research Tool http://smart.embl-heidelberg.de/[58]. To gain insight into the functional significance of the putatively selected sites, we also constructed the 3D structure of this protein and mapped selective sites onto it.

### Analysis of associations between ω and group size

A linear regression analysis was performed with R [59] to assess association between selection on TLR4 (terminal branch's ω (*dN/dS*) of the tree) and group sizes of cetaceans derived from May-Collado et al. [37]. Fourteen cetacean species with available data were included in this analysis. We calculated independent ω ratio for each branch of the tree by free-ratio model with CodeML in PAML version 4.

## Abbreviations

TLRs: Toll-like receptors; LPS: Lipopolysaccharides; MD-2: Myeloid differentiation factor 2; PRRs: Pattern recognition receptors; ORF: Open reading frame; CodeML: Codon-based maximum likelihood; SLAC: Single likelihood ancestor counting; REL: Random effects likelihood; FEL: Fixed effects likelihood; BEB: Bayes empirical Bayes; LRT: Likelihood ratio test; PAML: Phylogenetic Analysis by Maximum Likelihood; ML: Maximum Likelihood; NJ: Neighbor-Joining; EXT: Extracellular domain; TM: Transmembrane domain; CY: Cytoplasmic domain; MYA: Million years ago; 3D: Three-dimensional.

## Authors' contributions

GY conceived and designed the study, helped to perform data analyses and improve the manuscript. TS and SX performed the experiment and data analysis, and drafted the manuscript. XW helped to perform experiment. WY helped to perform data analysis. KZ helped to improve the manuscript. All authors read and approved the final manuscript.

## Supplementary Material

Additional file 1 **Table S1 Information about 17 representative cetaceans and some relative even-toed ungulates**. Click here for file

Additional file 2 **Table S2 Detailing the results of branch-site model analysis for positive selection at cetacean TLR4**. Click here for file

Additional file 3 **Table S3 Primers amplifying the complete ORF of representative cetaceans and some relative even-toed ungulates TLR4**. Click here for file
