# Supplementary material for: Adaptive evolution and functional constraint at TLR4 during the secondary aquatic adaptation and diversification of cetaceans
Source: BMC Evol Biol. 2012 Mar 24;12:39. doi: 10.1186/1471-2148-12-39 (PMC3384459; doi:10.1186/1471-2148-12-39)
Supplement: Additional file 1 — Table S1 Information about 17 representative cetaceans and some relative even-toed ungulates. [file 1471-2148-12-39-S1.PDF]

Table S1

Family	Species	Accession number	Reference
Lipotidae	Beiji (*Lipotes vexillifer*)	JN642614	This study
Platanistidae	Ganges susu (*Platanista gangetica*)	JN642616	This study
Physeteridae	Dwarf sperm whale (*Kogia simus*)	JN642613	This study
	Sperm whale (*Physeter catodon*)	AB500181	Shishido et al. (2010)
Monodontidae	Beluga whale (*Delphinapterus leucas*)	JN642610	This study
Phocoenidae	Indo-Pacific finless porpoise (*Neophocaena phocaenoides*)	JN642615	This study
Delphinidae	Indo-Pacific hump-backed dolphin (*Sousa chinensis*)	JN642617	This study
	Bottlenose dolphin (*Tursiops truncatus*)	JN642619	This study
	Killer whale (*Orcinus orca*)	AB492857	Shishido et al. (2010)
	Pacific white-sided dolphin (*Lagenorhynchus obliquidens*)	AB492856	Shishido et al. (2010)
	Striped dolphin (*Stenella coeruleoalba*)	JN642618	This study
	Long-beaked common dolphin (*Delphinus capensis*)	JN642611	This study
Balaenopteridae	Omura's whale (*Balaenoptera omurai*)	JN642609	This study
	Minke whale (*Balaenoptera acutorostrata*)	JN642608	This study
Suidae	Pig (*Sus scrofa*)	ENSSSCG00000005503	Shinkai and Uenishi, unpublished data
Bovidae	Water buffalo (*Bubalus bubalis*)	HM469969	Hariprasad et al. Unpublished data
Hippopotamidae	Hippopotamus (*Hippopotamus amphibius*)	JN642612	This study
