# Supplementary material for: Adaptive evolution and functional constraint at TLR4 during the secondary aquatic adaptation and diversification of cetaceans
Source: BMC Evol Biol. 2012 Mar 24;12:39. doi: 10.1186/1471-2148-12-39 (PMC3384459; doi:10.1186/1471-2148-12-39)
Supplement: Additional file 2 — Table S2 Detailing the results of branch-site model analysis for positive selection at cetacean TLR4. [file 1471-2148-12-39-S2.PDF]

Table S3

Tests for Positive Selection at Cetacean TLR4 with Branch-Site Models

Lineage[ψ]	Test 1 and 2	Models Compared	lnL	-2ln△L	Estimates of Parameters	*p* value	Positively Selected Sites (BEB Analysis) and Functional Information
		M1a	-6982.40		$p_0 = 0.59176$		
					$(p_1 = 0.40824)$		
					$\omega_0 = 0.06402$		
					$(\omega_1 = 1.00000)$		
a	1	Model A	-6979.68	5.44	$p_0 = 0.59658$	0.07	133K 0.723
					$(p_1 = 0.38666)$		139E 0.708 Adjacent to site involved in interaction with MD2
					$p_2 = p_3 = 0.01676$		149T 0.565
					$\omega_0 = 0.07056$		368T 0.576
					$(\omega_1 = 1\ \omega_2 = \infty)$		740D 0.790

	2	Model A ($\omega_2 = 1$)	-6982.38	5.40	$p_0 = 0.56360$<br>($p_1 = 0.38691$)<br>$p_2 + p_3 = 0.04948$<br>$\omega_0 = 0.06414$<br>($\omega_1 = \omega_2 = 1$)	0.02*	742R 0.697
Leading to *Delphinapterus leucas*	1	Model A	-6978.05	8.04	$p_0 = 0.59413$<br>($p_1 = 0.40392$)<br>$p_2 + p_3 = 0.00195$<br>$\omega_0 = 0.06505$<br>($\omega_1 = 1, \omega_2 = \infty$)	0.018	128P 0.875<br>302R 0.624 Adjacent to site involved in interaction with ligand binding<br>690Q 0.564
	2	Model A ($\omega_2 = 1$)	-6982.15	8.20	$p_0 = 0.48587$<br>(p_1=0.33062)<br>$p_2 + p_3 = 0.18351$<br>$\omega_0 = 0.06420$	0.004*	

					$(\omega_1 = \omega_2 = 1)$	
Leading to *Sus*	1	Model A	-6978.94	6.92	$p_0 = 0$ $(p_1 = 0)$	0.031
scrofa					$p_2 + p_3 = 1$	
					$\omega_0 = 0.03462$	
					$(\omega_1 = 1, \omega_2 = \infty)$	
	2	Model A	-6979.82	1.76	$p_0 = 0.00001$	0.185
		($\omega_2 = 1$）			$(p_1 = 0.00001)$	
					$p_2 + p_3 = 0.99999$	
					$\omega_0 = 0.02735$	
					$(\omega_1 = \omega_2 = 1)$	
e	1	Model A	-6979.25	6.3	$p_0 = p_1 = 0$	0.043
					$p_2 + p_3 = 1$	
					$\omega_0 = 0.03413$	
					$(\omega_1 = 1, \omega_2 = \infty)$	

	2	Model A	-6979.85	1.2	p_0 = 0.00057	0.273	
		(ω_2 = 1）			(p_1 = 0.00041)		
					p_2 + p_3=0.999		
					ω_0 = 0.02568		
					(ω_1 = ω_2 = 1)		
Leading to	1	Model A	-6981.9	1	p_0 = 0.58960	0.607	51E 0.550
Bubalus bubalis					(p_1 = 0.39683)		323M 0.504
					p_2 + p_3 = 0.01357		
					ω_0 = 0.06242		
					(ω_1=1,		
					ω_2=6.83696)		
	2	Model A	-6982.36	0.92	p_0 = 0.57938	0.337	
		(ω_2 = 1）			(p_1 = 0.39897)		
					p_2 + p_3 = 0.02165		

					$\omega_0 = 0.06072$		
					$(\omega_1 = \omega_2 = 1)$		
Leading to	1	Model A	-6981.02	2.76	$p_0 = 0.59211$	0.252	226S 0.674
Hippopotamus					$(p_1 = 0.39274)$		367N 0.702
amphibius					$p_2 + p_3 = 0.01515$		561V 0.645
					$\omega_0 = 0.06435$		
					$(\omega_1 = 1,$		
					$\omega_2 = 8.82086)$		
	2	Model A	-6982.39	2.74	$p_0 = 0.58717$	0.098	
		$(\omega_2 = 1)$			$(p_1 = 0.40410)$		
					$p_2 + p_3 = 0.00873$		
					$\omega_0 = 0.06339$		
					$(\omega_1 = \omega_2 = 1)$		
f	1	Model A	-6982.4	0	$p_0 = 0.59176$	1	

					($p_1 = 0.40824$)	
					$p_2 + p_3 = 0$	
					$\omega_0 = 0.06402$	
					($\omega_1 = \omega_2 = 1$)	
	2	Model A	-6982.4	0	$p_0 = 0.59176$	1
		($\omega_2 = 1$)			($p_1 = 0.40824$)	
					$p_2 + p_3 = 0$	
					$\omega_0 = 0.06402$	
					($\omega_1 = \omega_2 = 1$)	
g	1	Model A	-6982.4	0	$p_0 = 0.59176$	1
					($p_1 = 0.40824$)	
					$p_2 + p_3 = 0$	
					$\omega_0 = 0.06402$	
					($\omega_1 = \omega_2 = 1$)	

	2	Model A	-6982.4	0	$p_0 = 0.59176$	1	
		($\omega_2 = 1$)			($p_1 = 0.40824$)		
					$p_2 + p_3 = 0$		
					$\omega_0 = 0.06402$		
					($\omega_1 = \omega_2 = 1$)		
h	1	Model A	-6981.74	1.32	$p_0 = 0.37969$	0.517	99I 0.631
					($p_1 = 0.26463$)		196Q 0.560
					$p_2 + p_3 = 0.35568$		381S 0.615
					$\omega_0 = 0.05790$		682Q 0.567
					($\omega_1 = \omega_2 = 1$)		
	2	Model A	-6981.74	0	$p_0 = 0.37971$	1	
		($\omega_2 = 1$)			($p_1 = 0.26464$)		
					$p_2 + p_3 = 0.35565$		
					$\omega_0 = 0.05791$		

					$(\omega_1 = \omega_2 = 1)$	
i	1	Model A	-6982.4	0	$p_0 = 0.59175$	1
					$(p_1 = 0.40825)$	
					$p_2 + p_3 = 0$	
					$\omega_0 = 0.06401$	
					$(\omega_1 = \omega_2 = 1)$	
	2	Model A	-6982.4	0	$p_0 = 0.59176$	1
		$(\omega_2 = 1)$			$(p_1 = 0.40824)$	
					$p_2 + p_3 = 0$	
					$\omega_0 = 0.06402$	
					$(\omega_1 = \omega_2 = 1)$	
j	1	Model A	-6982.4	0	$p_0 = 0.59176$	1
					$(p_1 = 0.40824)$	
					$p_2 + p_3 = 0$	

					$\omega_0 = 0.06402$		
					$(\omega_1 = \omega_2 = 1)$		
	2	Model A	-6982.4	0	$p_0 = 0.59176$	1	
		($\omega_2 = 1$)			$(p_1 = 0.40824)$		
					$p_2 + p_3 = 0$		
					$\omega_0 = 0.06402$		
					$(\omega_1 = \omega_2 = 1)$		
l	1	Model A	-6981.52	1.76	$p_0 = 0$ $(p_1 = 0)$	0.415	247I 0.597
					$p_2 + p_3 = 1$		408I 0.602
					$\omega_0 = 0.06583$		419Q 0.816
					$(\omega_1 = 1, \omega_2 = \infty)$		430R 0.641
	2	Model A	-6982.4	1.76	$p_0 = 0.59176$	0.185	559V 0.621
		($\omega_2 = 1$)			$(p_1 = 0.40824)$		
					$p_2 + p_3 = 0$		

					$\omega_0 = 0.06402$	
					$(\omega_1 = \omega_2 = 1)$	
k	1	Model A	-6982.4	0	$p_0 = 0.59176$	1
					$(p_1 = 0.40824)$	
					$p_2 + p_3 = 0$	
					$\omega_0 = 0.06402$	
					$(\omega_1 = \omega_2 = 1)$	
	2	Model A	-6982.4	0	$p_0 = 0.59176$	1
		$(\omega_2 = 1$）			$(p_1 = 0.40824)$	
					$p_2 + p_3 = 0$	
					$\omega_0 = 0.06402$	
					$(\omega_1 = \omega_2 = 1)$	
b	1	Model A	-6982.4	0	$p_0 = 0.59176$	1
					$(p_1 = 0.40824)$	

					$p_2 + p_3 = 0$	
					$\omega_0 = 0.06402$	
					$(\omega_1 = \omega_2 = 1)$	
	2	Model A	-6982.4	0	$p_0 = 0.59176$	1
		$(\omega_2 = 1)$			$(p_1 = 0.40824)$	
					$p_2 + p_3 = 0$	
					$\omega_0 = 0.06403$	
					$(\omega_1 = \omega_2 = 1)$	
c	1	Model A	-6982.4	0	$p_0 = 0.59176$	1
					$(p_1 = 0.40824)$	
					$p_2 + p_3 = 0$	
					$\omega_0 = 0.06402$	
					$(\omega_1 = \omega_2 =1)$	
	2	Model A	-6982.4	0	$p_0 = 0.59176$	1

		$(\omega_2 = 1)$			$(p_1 = 0.40824)$		
					$p_2 + p_3 = 0$		
					$\omega_0 = 0.06402$		
					$(\omega_1 = \omega_2 = 1)$		
m	1	Model A	-6982.04	0	$p_0 = 0.20964$	1	62 K 0.718
					$(p_1 = 0.14545)$		
					$p_2 + p_3 = 0.64491$		
					$\omega_0 = 0.06156$		
					$(\omega_1 = \omega_2 = 1)$		
	2	Model A	-6982.04	0	$p_0 = 0.20985$	1	
		$(\omega_2 = 1)$			$(p_1 = 0.14559)$		
					$p_2 + p_3 = 0.64456$		
					$\omega_0 = 0.06156$		
					$(\omega_1 = \omega_2 = 1)$		

n	1	Model A	-6982.4	0	$p_0 = 0.59175$	1
					$(p_1 = 0.40825)$	
					$p_2 + p_3 = 0$	
					$\omega_0 = 0.06402$	
					$(\omega_1 = \omega_2 = 1)$	
	2	Model A	-6982.4	0	$p_0 = 0.59176$	1
		$(\omega_2 = 1)$			$(p_1 = 0.40824)$	
					$p_2 + p_3 = 0$	
					$\omega_0 = 0.06403$	
					$(\omega_1 = \omega_2 = 1)$	
o	1	Model A	-6982.4	0	$p_0 = 0.59176$	1
					$(p_1 = 0.40824)$	
					$p_2 + p_3 = 0$	
					$\omega_0 = 0.06402$	

					$(\omega_1 = \omega_2 = 1)$		
	2	Model A	-6982.4	0	$p_0 = 0.59176$	1	
		$(\omega_2 = 1)$			$(p_1 = 0.40824)$		
					$p_2 + p_3 = 0$		
					$\omega_0 = 0.06402$		
					$(\omega_1 = \omega_2 = 1)$		
p	1	Model A	-6981.54	1.72	$p_0 = 0$ $(p_1 = 0)$	0.423	84P 0.896
					$p_2 + p_3 = 1$		
					$\omega_0 = 0.06250$		
					$(\omega_1 = 1, \omega_2 = \infty)$		
	2	Model A	-6981.67	0.26	$p_0 = 0$ $(p_1 = 0)$	0.61	
		$(\omega_2 = 1)$			$p_2 + p_3 = 1$		
					$\omega_0 = 0.06238$		
					$(\omega_1 = \omega_2 = 1)$		

Leading to	1	Model A	-6982.4	0	$p_0 = 0.59176$	1
Platanista					$(p_1 = 0.40824)$	
gangetica					$p_2 + p_3 = 0$	
					$\omega_0 = 0.06402$	
					$(\omega_1 = \omega_2 = 1)$	
	2	Model A	-6982.4	0	$p_0 = 0.59177$	1
		($\omega_2 = 1$)			$(p_1 = 0.40823)$	
					$p_2 + p_3 = 0$	
					$\omega_0 = 0.06403$	
					$(\omega_1 = \omega_2 = 1)$	
Leading to	1	Model A	-6982.4	0	$p_0 = 0.59176$	1
Lipotes					$(p_1 = 0.40824)$	
vexillifer					$p_2 + p_3 = 0$	
					$\omega_0 = 0.06403$	

					($\omega_1 = \omega_2 = 1$)		
	2	Model A	-6982.4	0	$p_0 = 0.59176$	1	
		($\omega_2 = 1$)			($p_1 = 0.40824$)		
					$p_2 + p_3 = 0$		
					$\omega_0 = 0.06402$		
					($\omega_1 = \omega_2 = 1$)		
Leading to	1	Model A	-6982.31	0.18	$p_0 = 0.49279$	0.914	564F 0.621
Orcinus orca					($p_1 = 0.34143$)		
					$p_2 + p_3 = 0.16578$		
					$\omega_0 = 0.06241$		
					($\omega_1 = \omega_2 = 1$)		
	2	Model A	-6982.31	0	$p_0 = 0.49270$	1	
		($\omega_2 = 1$)			($p_1 = 0.34138$)		
					$p_2 + p_3 = 0.16592$		

					$\omega_0 = 0.06240$		
					$(\omega_1 = \omega_2 = 1)$		
Leading to *Lagenorhynchus obliquidens*	1	Model A	-6982.1	0.6	$p_0 = 0.51310$	0.741	192L 0.528
					$(p_1 = 0.35208)$		298G 0.575
					$p_2 + p_3 = 0.13482$		651F 0.613
					$\omega_0 = 0.06254$		
					$(\omega_1 = 1,$		
					$\omega_2 = 1.69006)$		
	2	Model A ($\omega_2 = 1$)	-6982.11	0.02	$p_0 = 0.45234$	0.888	
					$(p_1 = 0.31077)$		
					$p_2 + p_3 = 0.23689$		
					$\omega_0 = 0.06229$		
					$(\omega_1 = \omega_2 = 1)$		
Leading to	1	Model A	-6982.4	0	$p_0 = 0.59178$	1	

Sousa chinensis					(p_1 = 0.40822)	
					$p_2 + p_3 = 0$	
					ω_0 = 0.06404	
					($\omega_1 = \omega_2 = 1$)	
	2	Model A	-6982.4	0	p_0 = 0.59176	1
		($\omega_2 = 1$)			(p_1 = 0.40824)	
					$p_2 + p_3 = 0$	
					ω_0 = 0.06402	
					($\omega_1 = \omega_2 = 1$)	
Leading to	1	Model A	-6982.4	0	p_0 = 0.59176	1
Tursiops					(p_1 = 0.40824)	
truncatus					$p_2 + p_3 = 0$	
					ω_0 = 0.06402	
					($\omega_1 = \omega_2 = 1$)	

	2	Model A	-6982.4	0	$p_0 = 0.59176$	1	
		($\omega_2 = 1$)			($p_1 = 0.40824$)		
					$p_2 + p_3 = 0$		
					$\omega_0 = 0.06402$		
					($\omega_1 = \omega_2 = 1$)		
Leading to	1	Model A	-6980.62	3.56	$p_0 = 0$ ($p_1 = 0$)	0.169	118D 0.819
Stenella					$p_2 + p_3 = 1$		243H 0.809
coeruleoalba					$\omega_0 = 0.05802$		
					($\omega_1 = \omega_2 = 1$)		
	2	Model A	-6980.62	0	$p_0 = 0$ ($p_1 = 0$)	1	
		($\omega_2 = 1$)			$p_2 + p_3 = 1$		
					$\omega_0 = 0.05803$		
					($\omega_1 = \omega_2 = 1$)		
Leading to	1	Model A	-6982.4	0	$p_0 = 0.59175$	1	

Delphinus					(p_1 = 0.40825)		
capensis					$p_2 + p_3 = 0$		
					$\omega_0 = 0.06402$		
					($\omega_1 = \omega_2 = 1$)		
	2	Model A	-6982.4	0	$p_0 = 0.59176$	1	
		($\omega_2 = 1$）			(p_1 = 0.40824)		
					$p_2 + p_3 = 0$		
					$\omega_0 = 0.06402$		
					($\omega_1 = \omega_2 = 1$)		
Leading to	1	Model A	-6980.21	4.38	$p_0 = 0.44742$	0.112	24Q 0.785
Neophocaena					(p_1 = 0.30283)		28L 0.535
phocaenoides					$p_2 + p_3 = 0.24975$		178L 0.771
					$\omega_0 = 0.06050$		201Y 0.557
					($\omega_1 = 1$,		248N 0.831

					ω_2 = 3.63923)		525Q 0.551
	2	Model A	-6980.5	0.58	p_0 = 0.13632	0.446	542M 0.509
		(ω_2 = 1）			(p_1 = 0.09226)		556V 0.807
					$p_2 + p_3$ = 0.77142		608E 0.816
					ω_0 = 0.06045		
					($\omega_1 = \omega_2$ = 1)		
Leading to	1	Model A	-6982.34	0.12	p_0 = 0.56017	0.942	
Kogia simus					(p_1 = 0.38827)		
					$p_2 + p_3$ = 0.05156		
					ω_0 = 0.06120		
					($\omega_1 = \omega_2$ = 1)		
	2	Model A	-6982.34	0	p_0 = 0.56020	1	
		(ω_2 = 1）			(p_1 = 0.38829)		
					$p_2 + p_3$ = 0.05152		

					$\omega_0 = 0.06121$		
					$(\omega_1 = \omega_2 = 1)$		
Leading to	1	Model A	-6981.81	1.18	$p_0 = 0.55070$	0.554	87E 0.588
Physeter					$(p_1 = 0.37960)$		158G 0.565
catodon					$p_2 + p_3 = 0.0697$		506Q 0.547
					$\omega_0 = 0.05996$		548V 0.606
					$(\omega_1 = 1,$		593Q 0.543
					$\omega_2 = 3.75776)$		
	2	Model A	-6982.01	0.4	$p_0 = 0.47342$	0.527	
		$(\omega_2 = 1)$			$(p_1 = 0.32738)$		
					$p_2 + p_3 = 0.19921$		
					$\omega_0 = 0.05976$		
					$(\omega_1 = \omega_2 = 1)$		
Leading to	1	Model A	-6982.4	0	$p_0 = 0.59176$	1	

Balaenoptera acutorostrata					(p_1 = 0.40824)<br>$p_2 + p_3 = 0$<br>ω_0 = 0.06403<br>($\omega_1 = \omega_2 = 1$)	
	2	Model A ($\omega_2 = 1$)	-6982.4	0	p_0 = 0.59176<br>(p_1 = 0.40824)<br>$p_2 + p_3 = 0$<br>ω_0 = 0.06402<br>($\omega_1 = \omega_2 = 1$)	1
Leading to *Balaenoptera omurai*	1	Model A	-6982.4	0	p_0 = 0.59176<br>(p_1 = 0.40824)<br>$p_2 + p_3 = 0$<br>ω_0 = 0.06402<br>($\omega_1 = \omega_2 = 1$)	1

2	Model A	-6982.4	0	$p_0 = 0.59176$	1
	($\omega_2 = 1$)			($p_1 = 0.40824$)	
				$p_2 + p_3 = 0$	
				$\omega_0 = 0.06402$	
				($\omega_1 = \omega_2 = 1$)	

[ψ]: Lineages a to p corresponded to those labeled in Fig. 1.

*: $p < 0.05$ in the test 2.
