# Supplementary material for: Adaptive evolution and functional constraint at TLR4 during the secondary aquatic adaptation and diversification of cetaceans
Source: BMC Evol Biol. 2012 Mar 24;12:39. doi: 10.1186/1471-2148-12-39 (PMC3384459; doi:10.1186/1471-2148-12-39)
Supplement: Additional file 3 — Table S3 Primers amplifying the complete ORF of representative cetaceans and some relative even-toed ungulates TLR4. [file 1471-2148-12-39-S3.PDF]

Table S2

Primers in This Study

Primer Name	Primers Sequences (5'-3')	Amplification Target
TLR4-1F	TGCTTCATCTGCCTTGCTTG	Complete ORF of TLR4 in 11 Cetaceans
TLR4-1R	GGTGCCTGGTTCAATAAAGTC	Complete ORF of TLR4 in 11 Cetaceans
TLR4-2F	CTTTAGATTTGTCCCTGAACC	Complete ORF of TLR4 in 11 Cetaceans
TLR4-2R	TGTGAGAACAGCAACCCTTG	Complete ORF of TLR4 in 11 Cetaceans
TLR4-3F	CAAGGGTTGCTGTTCTCACA	Complete ORF of TLR4 in 11 Cetaceans
TLR4-3R	CTGACAAGTGGCATTCCTGA	Complete ORF of TLR4 in 11 Cetaceans
TLR4-4F	ACTACAGCATTTGCCAAGG	Complete ORF of TLR4 in 11 Cetaceans
TLR4-4R	TATGAAGATGATGCCAGCACG	Complete ORF of TLR4 in 11 Cetaceans
TLR4-5F	CCAGAGCCGATGGTGTATCT	Complete ORF of TLR4 in 11 Cetaceans
TLR4-5R	TGTTTATGCCCTCTGCATTG	Complete ORF of TLR4 in 11 Cetaceans

TLRHM4-1F	ATGTGAAATTAAGATTATTGA	Complete ORF of TLR4 in the Hippopotamus
TLRHM4-1R	AATTGGAATGCTGAAAATCC	Complete ORF of TLR4 in the Hippopotamus
TLRHM4-2F	CATTTAGATCTGAGCTTCAATGA	Complete ORF of TLR4 in the Hippopotamus
TLRHM4-2R	TTCATTCCGCACCCAGTCTTCAT	Complete ORF of TLR4 in the Hippopotamus
TLRHM4-3F	ATGAAGACTGGGTGCGGAATGAA	Complete ORF of TLR4 in the Hippopotamus
TLRHM4-3R	TCAGGTGGAGGTTGTCACTTCT	Complete ORF of TLR4 in the Hippopotamus
